# Supplementary material for: Influence of a change in activity regime on femoral bone architecture and failure behaviour
Source: PLoS One. 2024 Apr 29;19(4):e0297932. doi: 10.1371/journal.pone.0297932 (PMC11057758; doi:10.1371/journal.pone.0297932)
Supplement: Attachment — Submitted filename: PLoS One PONE-D-23-29003 R1 Response to Reviewers.pdf [file pone.0297932.s005.pdf]

Imperial College London

Department of Civil and Environmental Engineering
Skempton Building
South Kensington Campus
London SW7 2AZ

Email: andrew.phillips@imperial.ac.uk
Telephone: +44 (0)20 7594 6081

www.imperial.ac.uk/people/andrew.phillips

Dr Andrew Phillips
Reader in Structural Biomechanics

8 January 2024

Dear Ewa Tomaszewska,

Please find enclosed a revised version of the manuscript *Influence of a change in activity regime on femoral bone architecture and failure behaviour*.

The authors wish to thank you as the academic editor, and the reviewer for your consideration and comments on the paper which we believe have resulted in an improved manuscript, which we hope is now considered suitable for publication in PLoS One. We have included responses to the reviewer's comments below, where the comments are in blue, and our responses are in black.

Reviewer #1: The manuscript is generally well-written and presents a thoughtfully designed experiment. However, there are minor issues that the authors need to address.
I recommend introducing the scientific (medical) nomenclature for femur sections: diaphysis, metaphysis, and epiphysis. While these terms don't need to be used continuously throughout the manuscript, they are crucial, especially given the topic of bone fractures. The location of the fracture on the bone—whether on the diaphysis, metaphysis, or epiphysis—often determines the severity, implications, and health consequences.
We thank the reviewer for their appreciation of our work. We have amended the text throughout the manuscript to introduce medical nomenclature to identify specific anatomical regions of the femur, while retaining some terms which we feel will be more easily understood by an engineering as well as medical readership.

L29: The abbreviation "FE" should be explained upon its first usage.
We have added “finite element (FE)” as an explanation of “FE” here.

L33-34: Mechanostat is more appropriately described as a hypothesis or model rather than a principle.
We agree with the reviewer that it is more appropriate to describe the Mechanostat as a hypothesis rather than a principle and have amended the text here.

L77-78: For better readability, consider revising to: “Yield strain (y) in compression (c) and tension (t), and ultimate strains (u)…”
We have amended the text as suggested by the reviewer to improve readability.

L79: Please replace “y” with “c” in the ultimate tension formula.
We thank the reviewer for noticing the error and have corrected the subscript text.

L83: Use lowercase for "modulus".
We have corrected this.

L84: For clarity, consider writing as: E = 18.0 GPa.
We have amended the text as requested.

L95, other results, and Fig. 1: To enhance clarity, consider adding a decimal separator and a zero (".0") to integer values.
We have amended the text and the figure as requested.

L105-6: Please refer to these as the distal and proximal halves of the bone, indicating the respective diaphysis, metaphysis, and epiphysis.
We have amended the text to include anatomical terms, while retaining the original text which we believe is more readable for an engineering as well as a medical audience.

L116 and subsequent lines: "Diaphysis" is the precise anatomical term, preferable over "shaft". If the target audience includes technical readers such as engineers, they might find "shaft" more familiar. However, "diaphysis" remains the scientifically accurate term.
As with the comment above we have amended the text to use the term “diaphysis” while retaining the term “shaft” which we believe is more readable for a wider audience.

L122 and other lines: The term "bone resorption" is medical in nature. Given your focus on FE modeling, I suggest a rephrase like: "Most of the distal lateral trabecular bone exhibits changes (reductions) that may be interpreted as bone resorption...". Please consider implementing this correction in both the results and discussion sections, specifically the first instance it appears in each section. As I am not a native English speaker, I trust you can find a more optimal and appropriate way to phrase this if you choose to make the suggested changes.
While we feel that the term “bone resorption” is well known to readers familiar with computational modelling of bone adaptation we have amended the text to make clear that bone resorption describes a decrease in the volume of bone material. We have also amended the text to make clear that bone apposition describes an increase in the volume of bone material.

L148 and L153: Please amend to “Steen Jensen and Michaelsen’s classification”. Also, consider citing the original works available online 10.3109/17453677508989266 or 10.3109/17453678008990877)
We thank the reviewer for drawing our attention to these original works and have included these in the references, as well as amending the text.

L177: Correct to “delta_t” (without the h subscript).
We thank the reviewer for noticing the error and have amended the subscript text.

Tables 1 and 2: In the penultimate columns, please correct to the interval “[50,90]”. Clarify if presented intervals are left- or right-open.
We thank the reviewer for noticing the error and have amended the column heading in both tables. As the bin edges were implemented using the histogram function in MATLAB the intervals were left-closed and right-open. This has been clarified in the caption for the table. In practice due to the discretized nature of both the cross-sectional area of truss elements, and the thickness of shell elements, the percentage of values falling within the intervals are the same whether considered as left- or right-open.

Fig 1: Ensure figure captions stand alone in meaning. Add the reference to the original work by Phillips et al. and correct to “the original femur model [17] and each of the lower intensity activity regimes”.
We thank the reviewer for this suggestion and have amended the figure caption.

Fig 3-5: Amend to “c) the proximal and d) the distal epiphysis in … “
We thank the reviewer for the comment and have amended the captions for these figures.

Fig. 7: The caption for the y-axis appears to be missing.
We have moved the title of the graph so that it appears on the y-axis.

The authors also confirm with regards to the financial disclosure that the funders had no role in study design, data collection and analysis, decision to publish, or preparation of the manuscript. We would be happy to add this statement to the acknowledgement of the funding in the manuscript if required.

Once again, we thank the academic editor and the reviewer for their time in considering the manuscript.

Yours faithfully,

Andrew Phillips
